# The association between dream activity and alexithymia during pregnancy: A cross‐sectional study in a sample of pregnant women

**DOI:** 10.1111/jsr.14423

**Published:** 2024-12-10

**Authors:** Marta Spinoni, Serena Scarpelli, Ilaria Di Pasquale Benedetti, Carlotta Med, Paola Ciolli, Francesco Rech, Luigi De Gennaro, Caterina Grano

**Affiliations:** ^1^ Department of Psychology Sapienza University Rome Italy; ^2^ Department of Maternal and Child Health and Urological Sciences Sapienza University of Rome Rome Italy

**Keywords:** alexithymia, depression, dreaming, pregnancy

## Abstract

The gestational period is a sensitive time marked by significant changes that can affect women's sleep and dreaming processes, with an augmented frequency and recall of dreams suggesting that dreaming represents an adaptive mechanism of emotional regulation. This study investigates the relationship between pregnancy‐related variables, alexithymia, and depressive symptoms in influencing dream characteristics in women during the first trimester of pregnancy. A total of 118 pregnant women were recruited at the Obstetric Outpatient Service of an Italian University Hospital and completed the Mannheim Dream Questionnaire, the Toronto Alexithymia Scale‐20, and the Edinburgh Postnatal Depression Scale. Regression analysis, *t*‐test, and moderation analysis were conducted through Jamovi. Dream recall frequency was predicted by age, parity, and depressive symptoms. Nightmare frequency and lucid dream frequency were significantly predicted by depressive symptoms, while nightmare distress was predicted by an unplanned pregnancy. Alexithymia was linked to higher nightmare frequency and nightmare distress. Moderation analysis revealed that the presence of depressive symptoms predicted increased nightmare frequency only in women with higher levels of alexithymia. These findings highlight the role of emotional regulation in dreaming during pregnancy, particularly among women exhibiting alexithymic traits and depressive symptoms. Nightmare frequency may serve as an indicator of impaired emotional regulation, emphasising the need for targeted interventions to enhance emotional coping strategies in this population. Future research should examine the content of nightmares to further understand their implications for maternal mental health.

## 1 INTRODUCTION

The gestational period is a sensitive time marked by significant physical, biological, psychological, and hormonal transformations (Avise, 2013; Wadhwa, 2005), including increases in the production of oestrogens, progesterone, prolactin, and growth hormone. These changes also influence women's circadian patterns, affecting their sleep processes (Pengo et al., 2018; Sedov et al., 2018). Indeed, from the first trimester of pregnancy, women already exhibit elevated wakefulness after sleep onset, sleep‐related complaints, fragmented sleep, and diminished sleep quality compared with the pre‐pregnancy period (Hedman et al., 2002; Santiago et al., 2001), contributing to significant increases in the nightmare and dream recall frequency (Blake & Reimann, 1993; Lara‐Carrasco et al., 2013; Mancuso et al., 2008; Nowakowski et al., 2013; Scarpelli et al., 2024).

In line with the continuity hypothesis of dreaming (Domhoff, 2017), dream content may express an individual's waking thoughts, concerns, and emotions. Moreover, dreaming can reflect changes in people's lives and various aspects of the wakefulness experience (Scarpelli, Alfonsi, et al., 2022; Scarpelli, Nadorff, et al., 2022), including physical health and social roles. Also, increased dream recall may be provoked by a high rate of intrasleep awakenings (i.e. arousal‐retrieval theory, Koulack & Goodenough, 1976) and less deep sleep (i.e. activation hypothesis, Antrobus, 1991; Siclari et al., 2017; Scarpelli et al., 2017). It is important to emphasise that both the continuity hypothesis and the assumption that dreaming is related to high cortical activation do not attribute specific functions to dreaming. In fact, the continuity hypothesis considers dreams as a simple reflection of waking memory and other cognitive processes. Similarly, the activation hypothesis does not assign any functional role to dream recall. Instead, it views the successful retrieval of a dream trace upon awakening as merely the result of lighter sleep and a more desynchronised EEG pattern (Scarpelli et al., 2017; Siclari et al., 2017).

On the other hand, the high percentage of dreams reported by pregnant women and the high frequency of pregnancy‐related dream content may mirror the transition to a new identity as a mother (Ammaniti & Trentini, 2009; Sabourin et al., 2018). Indeed, pregnant women often report dreams in which they represent themselves and their imagined child, expressing concerns about motherhood and childbirth (Coo et al., 2014; Lara‐Carrasco et al., 2013). This is consistent with literature suggesting that critical life events or stressful conditions can lead to increased dream frequency and negative oneiric production (Gorgoni et al., 2022; Mathes et al., 2023). Although, again, this could be interpreted as a reflection of awake cognitive and emotional processes (Krippner et al., 1974; Schredl et al., 2016), more directly, the augmented frequency and recall of dreams by pregnant women could have a functional role in facilitating the preparation process for motherhood and childbirth (Cartwright, 2005, 2010; Mancuso et al., 2008; Scarpelli et al., 2019). Indeed, the literature has extensively suggested that dreaming may represent an adaptive mechanism of emotional regulation (Scarpelli et al., 2019; Sikka et al., 2022), especially during periods of changes and distress, allowing one to integrate the recent emotional experiences ultimately enhancing psychological well‐being (Cartwright, 2005, 2010; Mancuso et al., 2008). Namely, experiencing negative dreams or nightmares may help to prevent similar negative emotions from affecting wakefulness, thereby enhancing the individual's emotional well‐being during their waking hours.

In addition, the evolutionary theory of threat simulation proposes a functional role for nightmares: they simulate threatening or challenging situations, promoting the development of effective coping strategies for waking life (Revonsuo, 2000). Similarly, the social simulation theory posits that dreams, especially social interactions, may serve to simulate and rehearse social scenarios, improving the dreamer's ability to navigate relationships and social challenges (Tuominen et al., 2019). Consistently, the frequency of unpleasant dreams during pregnancy has been correlated with better outcomes in the peripartum period, including fewer obstetric complications, shorter labour duration, and less vulnerability to postpartum depression (Kron & Brosh, 2003; Mancuso et al., 2008). These findings suggest that dreaming, even when involving negative content, may serve adaptive functions in emotional processing, social simulation, and preparing for life challenges.

However, individuals experiencing higher levels of psychological distress report changes in how dreaming functions as a coping mechanism (Dagan et al., 2001; Parrello & Sommantico, 2022). The relationship between depressive symptoms and dreaming is far from straightforward, with research revealing mixed and sometimes contradictory findings. For instance, some studies suggest that dreams in depressed individuals tend to be shorter, more blank, and emotionally flat (Kron & Brosh, 2003). Additionally, while higher levels of depressive symptoms are associated with reduced dream recall, they also correlate with an increased frequency of nightmares (Barrett & Loeffler, 1992; Cartwright, 1986). In particular, nightmares are increasingly recognised as a significant factor in the relationship between depression and suicidal ideation, aligning with the continuity hypothesis (Domhoff, 2017; Faccini & Del‐Monte, 2024). Research highlights that depressed individuals experiencing frequent nightmares are at a heightened risk of suicidal thoughts and behaviours (Marinova et al., 2014; Tae et al., 2019). Additionally, underlying psychological factors such as rumination and difficulties in emotional regulation amplify this link, suggesting that nightmares may act as a mediating factor between these traits and suicidal behaviours (Rogers et al., 2017; Rufino et al., 2020). These findings reinforce the notion that nightmares in depressed individuals are not merely sleep disturbances but reflect deeper emotional struggles that warrant further clinical attention (Faccini & Del‐Monte, 2024).

Hence, the subjective experience of dreaming may mirror not only the wakeful emotional experiences but also the difficulties in emotional regulation strategies (Rufino et al., 2020; Wong & Yu, 2022). Consistently, depression is often associated with more emotional dysregulation, including higher levels of alexithymia (Honkalampi et al., 2020; Radetzki et al., 2021; Visted et al., 2018), and in depressive individuals, nightmares could also reflect failures in adaptive emotional strategies (Levin & Nielsen, 2007).

Alexithymia is characterised by an impaired ability to be aware of, explicitly identify, and describe one's feelings (Nemiah et al., 1976). In the general population, alexithymic traits, besides being correlated with sleep disturbances (self‐reported insomnia, daytime sleepiness, and recurrent nightmares; Nielsen et al., 2011; Murphy et al., 2018; Alimoradi et al., 2022), are also related to low dream frequency and a limited dream recall capacity (Lumley & Bazydlo, 2000; Lyvers et al., 2023). Notably, when individuals with alexithymia recall their dreams, they typically report peculiar experiences: dreams are often described as having explicit and primitive mental contents, lacking emotional depth, and are predominantly literally interpreted (De Gennaro et al., 2003; Formica et al., 2013). Moreover, it has been observed that the affective characteristics of alexithymic individuals, namely difficulty in identifying and describing emotions, are positively correlated with nightmare frequency (Bauermann et al., 2008; Lumley & Bazydlo, 2000) and with the tendency to have irregular, bizarre, and distressing dreams (Lumley & Bazydlo, 2000; Nielsen et al., 2011).

The existing literature on dream activity in pregnant women is notably heterogeneous and fragmented, and it is often characterised by multiple methodological limitations, including small sample sizes, unclear or insufficiently restrictive inclusion and exclusion criteria, and the occasional use of non‐standardised instruments for studying dreaming (Scarpelli et al., 2024). However, despite significant variation in study designs, the majority of research suggested that dreaming during pregnancy is marked by high dream recall and increased nightmare frequency (Blake & Reimann, 1993; Lara‐Carrasco et al., 2014; Nielsen & Paquette, 2007; Schredl et al., 2016), with dream content often reflecting pregnancy‐related themes (e.g. dreams depicting themselves as a mother or with babies/children, small animals, woman's pregnant body) and concerns (i.e. thoughts of delivery and childbirth, more manifestations of apprehensions in dreams, medical elements) that parallel their waking experiences, (Blake & Reimann, 1993; Dagan et al., 2001; Gül and Solt, 2021; Kalmbach et al., 2019; Krippner et al., 1974; Lara‐Carrasco et al., 2013; Nielsen & Paquette, 2007; Schredl et al., 2016; Van et al., 2023).

Notably, most of the studies did not consider pregnancy‐related psychological aspects (i.e. peripartum depression), even considering that these are prevalent in pregnancy and during the postpartum period and that they may importantly influence the dreaming processes (Lara‐Carrasco et al., 2013; Sabourin et al., 2018). In addition, to date, no studies have investigated the association between dream activity and emotional regulation, including alexithymic traits, in pregnant women. Furthermore, the majority of research on dreaming in pregnancy has focussed on the third trimester, resulting in a notable absence of data concerning dream activity during the first trimester. Investigating dream activity during this early gestational period is particularly important, as it marks the onset of physiological and hormonal changes that could influence dreaming and may differentiate this population from non‐pregnant individuals. Moreover, women in the late third trimester demonstrate fewer specific representations of babies or children in their dreams compared with those in the early trimesters, suggesting that dream patterns may evolve distinctly over pregnancy, making first‐trimester dream activity especially informative for study (Lara‐Carrasco et al., 2013).

To fill these gaps, the aims of the present study are the following. (I) Firstly, we intend to identify which pregnancy‐related factors may predict state‐like dream characteristics during the first trimester of pregnancy, considering dream frequency, nightmare frequency, lucid dream frequency, nightmare distress, and dream‐related emotional intensity. Based on the continuity hypothesis, which suggests that waking emotional and physiological states carry over into dream content, we hypothesise that both poor sleep quality and depressive symptoms during pregnancy will influence dream experiences. Specifically, poor sleep quality may predict greater dream recall and an increased frequency of nightmares, while depressive symptoms may lead to more frequent and distressing nightmares due to the amplification of negative emotional states.

(II) Second, we aim to investigate differences in dream characteristics between pregnant women who exhibit alexithymic traits and those who do not, as well as to examine whether alexithymic traits moderate the relationship between depression, sleep quality, and dream characteristics. Given the threat simulation theory, which posits that dreams, particularly nightmares, serve to simulate threatening experiences, we hypothesise that pregnant women with alexithymic traits—who typically experience increased emotional stress and have limited emotional insight—may report higher nightmare frequency and greater nightmare distress. Additionally, according to the emotion regulation hypothesis, which suggests that dreams may serve as a means of processing emotions, we expect that the elevated stress levels associated with alexithymia may lead to increased dream activity as an attempt to regulate emotions during pregnancy. Consequently, alexithymia may heighten the impact of depression and poor sleep quality on nightmare occurrence and distress, leading to more intense or distressing dream experiences for these women.

## 2 METHODS

### 2.1 Procedures and participants

The study is part of a research project promoted by the Sapienza University of Rome. Recruitment was performed from April 2023 to December 2023 at the Obstetric Outpatient Service of the Policlinico Umberto I University Hospital of Rome and through different social media platforms via Qualtrics platform (Qualtrics@, Provo, UT). The recruitment considered the following inclusion criteria: age equal to or greater than 18 years; the ability to understand and complete questionnaires in the Italian language; being in a state of pregnancy (max. 17 weeks) at the time of questionnaire completion. All participants took part voluntarily and were not remunerated. After explaining the study and obtaining online informed consent, women were asked to complete an online questionnaire.

Ethical authorisation was obtained from the Institution Review Board of the Psychology Department, Sapienza University of Rome (Prot N. 0002518). The study was conducted according to the Code of Ethics of the Italian Psychological Association and the American Psychological Association.

Participants included 118 pregnant women aged between 19 and 45 (M = 32.2 SD = 5.54). The majority (94.1%) of the sample was Italian, with only 23.7% having experienced one or more previous pregnancies. The level of education was assessed in terms of years of schooling, revealing an average of 14.9 years of education among participants (SD = 3.91). Further demographic characteristics of the sample are shown in Table 1 (see the Results section).

**TABLE 1:** Sociodemographic characteristics of the participants in the study (*n* = 118).

Variable	Mean ± DS or *N* (%)
Age	32.2 ± 5.54
Education (years)	14.9 ± 3.91
Nationality	
Italian	111 (94.1)
Other	7 (5.9)
Gestational month	
First month	6 (5.1)
Second month	37 (31.4)
Third month	75 (63.5)
Parity status	
Primiparous	90 (76.3)
Multiparous	28 (23.7)
Miscarriage	
Yes	28 (23.7)
No	90 (76.3)
Pregnancy complications	
Yes	24 (22.2)
No	84 (77.8)
Planned pregnancy	
Yes	87 (73.7)
No	31 (26.3)
Sleep quality	
Good sleeper	80 (67.8)
Bad sleeper	38 (32.2)
Alexithymia	
TAS > cut‐off	43 (36.4)
TAS TOT	46.4 ± 12.8
Depression	
EPDS > cut‐off	43 (36.4)
EPDS TOT	7.93 ± 5.24
Dream variables	
Dream frequency	4.15 ± 1.59
Nightmare frequency	2.60 ± 2.27
Lucid dream frequency	2.49 ± 2.24
Nightmare distress	1.43 ± 1.06
Emotional intensity	1.95 ± 1.00

### 2.2 Instruments

For data collection, various questionnaires were administered. Firstly, women were asked to report sociodemographic data (age, weight, height, parity status, education, marital status, and nationality), along with pregnancy‐related information (months of gestation, presence of pathologies during pregnancy).

The Italian Mannheim Dream Questionnaire (MADRE; Schredl et al., 2014; Settineri et al., 2019) was used to collect information on the intensity of nightmares. It consists of a total of 20 items, each referring to a specific dimension of dream experiences. According to the objectives, the current study focussed on one item concerning dream recall frequency (*How often have you recalled your dreams recently?*), nightmare frequency (*How often have you experienced nightmares recently?*), lucid dream frequency (*How often do you experience so‐called lucid dreams?*), nightmare distress (*If you currently experience nightmares, how distressing are they to you?*), and emotional intensity (*How intense are your dreams emotionally?*). Responses are provided using an ordinal scale with five options, ranging from 0 (not at all distressing) to 4 (very distressing), reflecting varying levels of intensity and frequency.

To test the subject's level of alexithymia, use was made of the Italian version of the 20‐Toronto Alexithymia Scale (TAS‐20) (Bressi et al., 1996; Taylor et al., 1992). This scale consists of 20 items assessing the difficulty in identifying feelings (DIF subscale; e.g. item: *I have feelings that I can't quite identify*), difficulty in describing feelings (DEF subscale, e.g. item *It is difficult for me to find the right words for my feeling*), and the externally oriented style of thinking (EOT subscale, e.g. item *I prefer to analyse problems rather than just describe them*). Responses to each question are scored on a 5‐point rating scale ranging from 1 (strongly disagree) to 5 (strongly agree), with higher scores indicating higher levels of alexithymia. In the current sample, Cronbach's alpha was 0.87 for the total score, 0.86 for the DIF, 0.79 for the DEF, and 0.60 for the EOT. The total scores of the TAS‐20 were categorised into two groups as follows: a score of at least 52 indicated alexithymia, and less than 52 no alexithymia (Bressi et al., 1996).

Sleep quality was evaluated through one item of the Italian version of the Pittsburgh Quality Index (Buysse et al., 1989; Curcio et al., 2013), the wording “*During the past month, how would you rate your sleep quality overall*.” Response rates are scored on a 4‐point rating scale from 1 (very good) to 4 (very bad). As it consists of a single item, this variable was dichotomised [0= good sleepers (score <2); 1= poor sleepers (score >=2)].

Finally, to evaluate depressive symptoms, the Italian version of The Edinburgh Postnatal Depression Scale (EPDS; Cox et al., 1987; Benvenuti et al., 1999) was administered. The scale consists of 10 items designed to assess perinatal depression symptoms referring to the preceding week. An example of an item is “*I have been so unhappy that I have had difficulty sleeping*”, with a 4‐point response scale from 0 (no, not at all) to 3 (yes, most of the time). Following the literature, we dichotomised the total score of the EPDS as follows: 0 = absence of significant depressive symptoms (score <10), 1 = presence of depressive symptoms (score >=10). In the current sample, Cronbach's alpha was 0.91.

### 2.3 Statistical analysis

All analyses were conducted using the statistical software Jamovi (The Jamovi Project, 2019).

Descriptive statistics are expressed as mean (M) ± standard deviation (SD) or as the number of participants (N) with the percentage in parenthesis.

The distribution of continuous variables was investigated using descriptive statistics, considering skewness and kurtosis (values of skewness and kurtosis ranging between –1 and +1 were considered acceptable). After verifying the skewness and kurtosis values of the variables of interest, five ordinal regressions were conducted to ascertain which pregnancy‐related variables predict the various state‐like dream variables (Hypothesis I): frequency of dream recall (DRF), frequency of nightmares (NMF), frequency of lucid dreams (LDF), nightmare distress (ND), and emotional intensity of dreams (IE). In each regression model, the following covariates were included based on the relevant literature: age, parity, presence of pregnancy‐related pathologies, planned pregnancy, sleep quality, and presence of depression. Additionally, in the regression models for lucid dreams and nightmare distress, we also controlled for levels of nightmare frequency. Variance inflation factor (VIF) values and tolerance values were generated for the regression model to assess multicollinearity and to test that the assumption was not violated (VIF < 10 and tolerance >0.1). The significance level was set to *p* < 0.05, with a precise *p*‐value reported for all test results.

In line with Hypothesis II, we employed the non‐parametric Mann‐Whitney U test (independent samples comparison) to compare alexithymic and non‐alexithymic women in dream characteristics. The sample was divided into alexithymic and non‐alexithymic women based on the cutoff established in the literature for TAS (Bagby et al., 2020), and the following dependent variables were considered: dream recall frequency, nightmare frequency, lucid dreams frequency, nightmare distress, and emotional intensity.

For the characteristics that were significantly different between alexithymic and non‐alexithymic pregnant women, we further investigated correlation (Spearman's ρ) among these variables and alexithymia subscales (difficulty in identifying feelings, difficulty in describing feelings, external‐oriented thinking).

Finally, to examine whether the presence of alexithymic traits may influence the relationship between predictors of dreaming (i.e. depression and sleep quality) and state‐like dream variables, moderation analyses were conducted through an ordinal regression inserting the interaction between TAS and EPDS scores, while controlling for covariates (age, parity, miscarriage, pregnancy complication, planned pregnancy, sleep quality).

## 3 RESULTS

Table 1 shows the sociodemographic and clinical characteristics of the sample. Prepartum depressive scores ranged from 0 to 25, with 36.4% of women above the cut‐off. Alexithymia scores ranged from 22 to 75, with 36.4% of women above the cut‐off of 52.

### 3.1 Pregnancy‐related characteristics associated with dreaming (Hypothesis I)

In line with Hypothesis I, we conducted five ordinal regression analyses to examine which pregnancy‐related variables predict the different state‐like dream variables.

Table 2 presents the results of the ordinal regression model that examined the association between pregnant‐related characteristics and dream frequency. The results indicated that age (β = −0.07, *p* = 0.036), parity (β = 1.15, *p* = 0.014), pregnancy complication (β = 0.99, *p* = 0.044), and depressive symptoms (β = 1.18, *p* = 0.002) were significantly associated with dream recall frequency. The model accounted for 6% of the variance in dream recall frequency. Thus, being younger, having experienced previous pregnancy, reporting pregnancy complications, and the presence of depressive symptoms were indicative of heightened levels of dream recall frequency. Table 3 presents the results of the ordinal regression model that examined the association between pregnant‐related characteristics and nightmare frequency. The results indicated that sole depressive symptoms (β = 1.15, *p* = 0.002) were significantly positively associated with nightmare frequency. The model accounted for 3% of the variance.

**TABLE 2:** Regression analyses with dream frequency as the dependent variable.

Variable	Estimate	SE	Sign	95% confidence interval	
				Lower bound	Upper bound
Age	**−0.07**	**0.03**	**0.036**	**0.866**	**0**.**995**
Parity	**1.15**	**0.47**	**0.014**	**1.273**	**8.013**
Miscarriage	**−**0.78	0.45	0.087	0.184	1.115
Pregnancy complication	**0.99**	**0.49**	**0.044**	**1**.**036**	**7.217**
Planned pregnancy	**−**0.55	0.43	0.201	0.250	1.339
Sleep quality	**−**0.19	0.42	0.643	0.361	1.878
Depressive symptoms	**1.18**	**0.38**	**0.002**	**1.569**	**7.024**

*Note*: Bold font indicates statistical significance (*p* < 0.05). Model significance: R^2^ = 0.059; χ^2^(7) = 21.5, *p* = 0.003.

**TABLE 3:** Regression analyses with nightmare frequency as dependent variable.

Variable	Estimate	SE	Sign	95% confidence interval	
				Lower bound	Upper bound
Age	−0.01	0.03	0.755	0.928	1.05
Parity	−0.26	0.45	0.569	0.312	1.89
Miscarriage	0.54	0.44	0.219	0.729	4.11
Pregnancy complication	0.19	0.46	0.664	0.498	3.03
Planned pregnancy	0.19	0.41	0.629	0.542	2.75
Sleep quality	0.41	0.42	0.329	0.660	3.44
Depressive symptoms	**1.15**	**0.37**	**0.002**	**1.541**	**6.63**

Notes: Bold font indicates statistical significance (*p* < 0.05). Model significance: R^2^= 0.035; χ^2^(7) = 14.1, *p* = 0.049.

Table 4 presents the results of the ordinal regression model that examined the association between pregnant‐related characteristics and lucid dream frequency. The results indicated that sleep quality (β = 0.83, *p* = 0.045) and nightmare frequency (β = 0.21, *p* = 0.021) were significantly positively associated with lucid dream frequency. The model accounted for 6% of the variance. Thus, being a bad sleeper and reporting higher nightmare frequency is associated with higher lucid dream frequency.

**TABLE 4:** Regression analyses with lucid dreams frequency as dependent variable.

Variable	Estimate	SE	Sign	95% confidence interval	
				Lower bound	Upper bound
Age	−0.05	0.03	0.148	0.885	1.02
Parity	0.76	0.45	0.089	0.891	5.14
Miscarriage	0.68	0.45	0.131	0.816	4.78
Pregnancy complication	0.29	0.47	0.540	0.523	3.43
Planned pregnancy	−0.82	0.44	0.059	0.183	1.02
Sleep quality	**0.83**	**0.42**	**0.045**	**1.023**	**5.24**
Depressive symptoms	0.55	0.38	0.153	0.917	3.70
Nightmare frequency	**0.21**	**0.09**	**0.021**	**1.034**	**1.48**

*Note*: Bold font indicates statistical significance (*p* < 0.05). Model significance: R^2^ = 0.062; χ^2^(7) = 24.7, *p* = 0.002.

Table 5 presents the results of the ordinal regression model that examined the association between pregnant‐related characteristics and nightmare distress. The results indicated that beyond nightmare frequency (β = 0.24, *p* = 0.009), sole unplanned pregnancies (β = 1.09, *p* = 0.025) were significantly associated with nightmare distress. Thus, the absence of planning for pregnancy predicted higher nightmare distress. The model accounted for 8% of the variance.

**TABLE 5:** Regression analyses with nightmare distress as the dependent variable.

Variable	Estimate	SE	Sign	95% confidence interval	
				Lower bound	Upper bound
Age	0.04	0.03	0.329	0.964	1.12
Parity	−0.77	0.50	0.132	0.168	1.25
Miscarriage	0.62	0.47	0.191	0.733	4.72
Pregnancy complication	0.36	0.53	0.899	0.350	2.82
Planned pregnancy	**1.09**	**0.48**	**0.025**	**1.156**	**7.85**
Sleep quality	0.48	0.46	0.292	0.659	4.00
Depressive symptoms	−0.02	0.41	0.968	0.438	2.20
Nightmare frequency	**0.24**	**0.09**	**0.009**	1.**063**	**1.53**

*Note*: Bold font indicates statistical significance (*p* < 0.05). Model significance: R^2^ = 0.085; χ^2^(7) = 22.9, *p* = 0.003.

The last ordinal regression model that examined the association between pregnant‐related characteristics and dream‐related emotional intensity indicated that the model was not significant (χ^2^ = 12.6, *p* = 0.125).

### 3.2 Dream characteristics differences between alexithymic and non‐alexithymic women (Hypothesis II)

**FIGURE 1:**
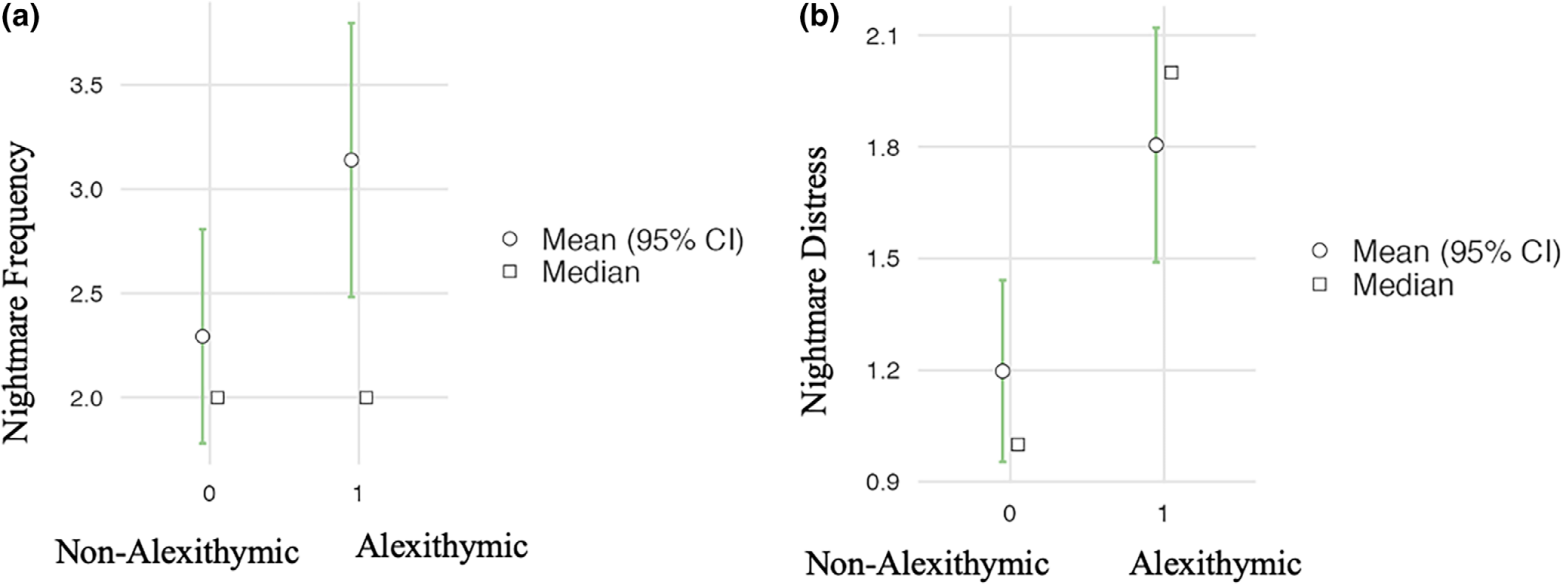
Differences in nightmare frequency (a) and nightmare distress (b) scores between alexithymic and non‐alexithymic group.

Table 6 presents the results of the Mann‐Whitney test conducted to investigate the differences in dream characteristics between alexithymic (TAS>=52) and non‐alexithymic (TAS<52) women (Hypothesis II). The results showed a significant difference in nightmare frequency (z = 1,215 *p* = 0.024) and nightmare distress scores (z = 904, *p* = 0.003) between alexithymic and non‐alexithymic women. Specifically, women who reported alexithymic traits present both higher nightmare frequency (Figure 1a) and nightmare distress (Figure 1b) scores.

**TABLE 6:** Differences in dream characteristics between alexithymic and non‐alexithymic.

Variable	Alexithymic (*n* = 43)	Non‐alexithymic (*n* = 75)	*U* Mann‐Whitney	Effect size	*p* value
Dream frequency	4.50	4.00	1460	.073	0.506
Nightmare frequency	2.00	2.00	**1215**	.**246**	**0.024**
Lucid dreams frequency	3.00	2.00	1467	.078	0.476
Nightmare distress	2.00	1.00	**904**	.**332**	**0.003**
Emotional intensity	2.00	2.00	1409	.126	0.231

*Note*: Bold font indicates statistical significance (*p* < 0.05).

Since nightmare frequency and nightmare distress were significantly different between alexithymic and non‐alexithymic pregnant women, we further investigated the correlation (Spearman's ρ) among these variables and alexithymia subscales. Table 7 reports the correlations between the three TAS domains (difficulty in identifying feelings, difficulty in describing feelings, externally orientated thinking), nightmare frequency, and nightmare distress. The difficulty in identifying feelings is positively correlated with both nightmare frequency (ρ = 0.28, *p* = 0.009) and nightmare distress (ρ = 0.36, *p* < 0.001). Conversely, difficulty in describing feelings is only positively related to nightmare distress (ρ = 0.22, *p* = 0.012).

**TABLE 7:** Correlation analysis of the variables of interest.

	1.	2.	3.	4.	5.
1. Difficulty in identifying feelings	‐				
2. Difficulty in describing feelings	0.67***	‐			
3. Externally orientated thinking	0.33***	0.44***	‐		
4. Nightmare frequency	0.28**	0.17	−0.02	‐	
5. Nightmare distress	0.36***	0.22*	‐0.03	0.35**	‐

*Note*: *: *p* < 0.05; **: *p* < 0.01; ***: *p* < 0.001.

**FIGURE 2:**
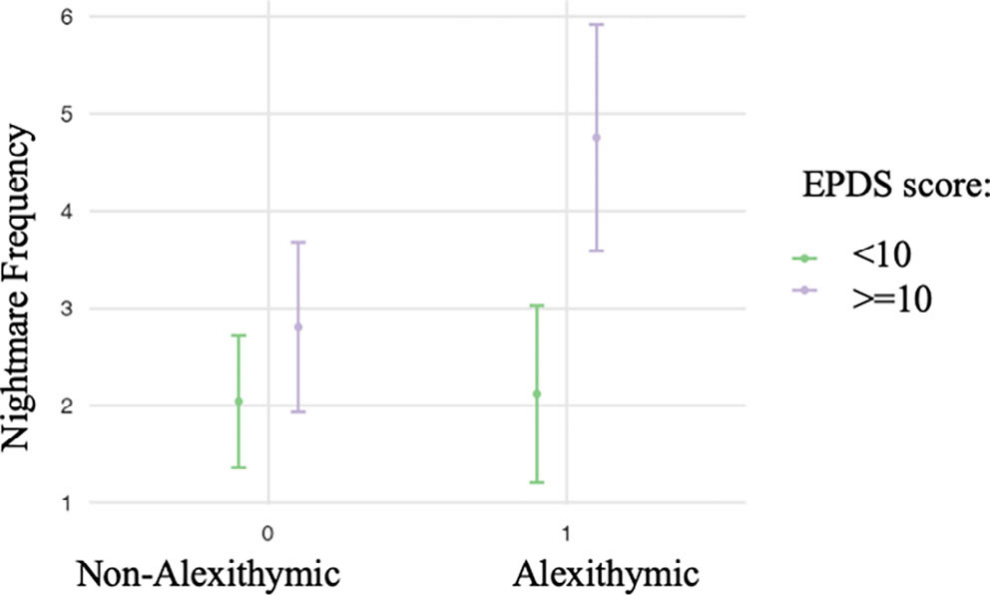
The interaction between the presence of alexithymia and depressive symptoms in predicting nightmare frequency.

Table 8 shows the results of the regression model examining the significant interaction between the presence of alexithymic traits and depressive symptoms in predicting nightmare frequency, controlling for covariates. In Step 1, all covariates were added to the model, and the model was not statistically significant (F = 0.73, *p* = 0.624). In Step 2, the presence of depressive symptoms was inserted, showing a positive association between depression and nightmare distress (β = 1.44, *p* = 0.002). In Step 3, the presence of alexithymia was included in the model, showing no association between alexithymia presence and nightmare frequency (β = 0.75, *p* = 0.129). Finally, in Step 4, the interaction between alexithymia and depression was added and remained the sole predictor of the nightmare distress (β = 1.87, *p* = 0.044). The final model accounted for 19% of the variance in nightmare frequency. The results showed that the presence of depressive symptoms was associated with nightmare frequency only in women with TAS >=52 (Figure 2).

**TABLE 8:** Regression analysis with nightmare frequency as dependent variable.

	Variable	R^2^	Beta (β)	F	Sign
Step 1		0.04		0.73	0.624
	Age		−0.02		0.632
	Parity		−0.34		0.564
	Miscarriage		0.63		0.282
	Pregnancy complication		0.45		0.450
	Planned pregnancy		0.13		0.813
	Sleep quality		0.54		0.303
Step 2		0.14		2.19	**0.041**
	Age		−0.01		0.704
	Parity		−0.31		0.587
	Miscarriage		0.61		0.271
	Pregnancy complication		0.31		0.585
	Planned pregnancy		0.08		0.885
	Sleep quality		0.58		0.244
	Depressive symptoms		**1.44**		**0.002**
Step 3		0.16		2.24	**0.031**
	Age		−0.01		0.884
	Parity		−0.26		0.639
	Miscarriage		0.55		0.324
	Pregnancy complication		0.36		0.529
	Planned pregnancy		−0.21		0.696
	Sleep quality		0.55		0.268
	Depressive symptoms		**1.45**		**0**.**002**
	Alexithymia		0.75		0.129
Step 4		0.19		2.52	
	Age		0.01		0.954
	Parity		−0.37		0.495
	Miscarriage		−0.38		0.374
	Pregnancy complication		0.34		0.551
	Planned pregnancy		−0.36		0.512
	Sleep quality		0.62		0.204
	Depressive symptoms		0.76		0.169
	Alexithymia		0.08		0.898
	Alexithymia*Depression		**1.87**		**0.044**

*Note*: Bold font indicates statistical significance (*p* < 0.05). Step 1: F(6,111) = 0.73, *p* = 0.624, Adj. R^2^ = −0.02; Step 2: F(7,110) = 2.19, *p* = 0.041, Adj. R^2^ = 0.08; Step 3: F(8,109) = 2.24, *p* =0.031, Adj. R^2^ = 0.09; Step 4: F(9,108) = 2.52, *p* = 0.012. Adj. R^2^ = 0.11.

Model 1‐ Model 2 R^2^ change = 0.09, *p* = 0.002; Model 2‐Model 3 R^2^ change = 0.02 *p* = 0.129. Model 3 ‐ Model 4 R^2^ change = 0.04, *p* = 0.044.

## 4 DISCUSSION

The first aim of this study was to explore which pregnancy‐related variables in women during the first trimester were significantly associated with dream characteristics. Our findings suggested that dream recall frequency was predicted by age, parity, and prepartum depressive symptomatology. Additionally, the frequency of nightmares was predicted by depressive symptoms; nightmare distress was predicted by an unplanned pregnancy, and lucid dreams frequency was predicted by sleep quality and nightmare frequency. Regarding the relationship between alexithymia and dream experiences, the findings showed that difficulty in identifying one's emotions was positively correlated with both the frequency of nightmares and nightmare distress. Finally, alexithymia moderated the relationship between depression and nightmare frequency, indicating that nightmare frequency was higher in the group of depressed women who were also alexithymic. This group of women probably uses nightmares as a regulatory process in the absence of other regulatory functions (i.e. difficulty in identifying feelings). On the contrary, the group of depressed women who do not have difficulties in identifying feelings (i.e. do not lack an emotional regulatory process) reported lower levels of nightmare frequency.

### 4.1 Predictors of dream characteristics in pregnant women

In line with our first hypothesis, the findings suggested that during the first trimester, both individual variables (e.g. age) and specific pregnancy‐related variables (e.g. parity, planned pregnancy, and perinatal depressive symptoms) significantly predict pregnant women's dream experiences. Our results indicate that younger women report a higher frequency of dream recall, which aligns with prior research showing similar patterns in both healthy and clinical subjects (Giambra et al., 1996; Nielsen, 2012; Schredl et al., 2014) and clinical subjects (e.g. in long‐COVID patients, Scarpelli et al., 2022). Although the literature suggests that females increase access to their dreams at a younger age, the exact mechanisms responsible for the age‐related decline in dream recall remain uncertain (Nielsen, 2012).

Multiparous women recall dreams more frequently than primiparous women. Although the literature on the effect of parity on dreaming is limited, this finding is not entirely consistent with prior research. Previous studies indicated primiparous women report more dream content related to their future child than multiparous women (Sabourin et al., 2018; Schredl et al., 2016). Additionally, during the postpartum period, primiparous women demonstrated a higher frequency of dream recall compared with multiparous women (Nielsen & Paquette, 2007). It should be noted, however, that these studies evaluated women at the end of pregnancy, whereas in our study, most of the sample was in the first trimester. At the beginning of pregnancy, maternal representations related to caring for a newborn and associated concerns might be reactivated, in line with the continuity hypothesis (Domhoff, 2017). Indeed, it is well known that significant changes and stressful events can elicit increased dream production, and increased dreaming has been reported following stressful events, including divorces, the COVID‐19 pandemic, and earthquakes (Cartwright et al., 2006; Hartmann et al., 2001; Propper et al., 2007; Scarpelli, Alfonsi, et al., 2022; Scarpelli, Nadorff, et al., 2022). Furthermore, it is also important to consider that frequent urination is a common symptom during the first trimester of pregnancy, leading to more fragmented sleep and increased nighttime awakenings, and this may explain the higher dream frequency in our sample (Delgado & Louis, 2022). Unfortunately, we did not measure nocturnal awakening frequency, and therefore, we cannot establish its different influence on dream recall in primiparous and multiparous women. However, recent evidence indicates that multiparous women experience poorer sleep quality compared with primiparous women (e.g. Huong et al., 2019).

Another notable finding is that the presence of depressive symptoms predicts both dream frequency and the occurrence of nightmares. Concerning the relationship between depression and dream frequency, it is noteworthy that the association between depressive symptoms and nightmare frequency appears more robust than those between depression and dream recall frequency. Numerous studies have documented that psychological distress is associated with increased nightmare frequency (Forest et al., 2023), including depressive symptomatology (Agargun et al., 2007; Akkaoui et al., 2020; Geoffroy et al., 2022). Considering the peripartum period, few studies focussed on the relationship between prenatal depressive symptoms and dream characteristics. One study reported that depression levels during pregnancy were higher in women reporting masochistic dreams (i.e. bad dreams with themes of self‐inflicted suffering or passively enduring unpleasant experiences; Mancuso et al., 2008), and another study highlighted that women with pregnancy‐related insomnia reported more frequent nightmares and depressive symptoms (Wołyńczyk‐Gmaj et al., 2017). Some authors also suggested that dream content during pregnancy can reflect underlying psychological states and may serve as early indicators of subsequent postpartum psychological diseases, including postpartum depression (PPD; Kron & Brosh, 2003). In particular, women without PPD have had a greater frequency of masochistic dreams during pregnancy compared with other dream content, while women with PPD reported the opposite pattern (Kron & Brosh, 2003). Moreover, women without PPD exhibited a higher frequency of dreams manifesting apprehension compared with those who later developed PPD, indicating a possible preparatory function of this type of dreams and nightmares in anticipating future challenges with the child (Kron & Brosh, 2003). This hypothesis is in line with existing literature on the impact of stressful life events or conditions on dream frequency and content (Gorgoni et al., 2022; Mathes et al., 2023). Specifically, in the context of pregnancy, it also aligns with findings that pregnant women often experience an increased frequency of bad dreams, which may serve a functional role in preparing them for motherhood and childbirth (Cartwright, 2005, 2010; Lara‐Carrasco et al., 2013; Mancuso et al., 2008; Scarpelli et al., 2019). Indeed, the literature extensively suggested that dreaming has an adaptive mechanism for emotional regulation (Rufino et al., 2020; Scarpelli et al., 2019; Sikka et al., 2022), particularly during periods of significant change and stress, with the function of integrating recent emotional experiences, ultimately enhancing psychological well‐being (Cartwright, 2005, 2010; Mancuso et al., 2008).

The regulatory function of oneiric activity can also explain the association between nightmare and lucid dream frequency that emerged in our findings, potentially reflecting an adaptive process wherein increased nightmare frequency could foster the development of lucid dreaming as a natural self‐regulatory response. Consistently, recent research suggests that lucid dreaming might serve as a coping mechanism, enabling individuals to confront and manage the distressing elements of their dreams (Scarpelli et al., 2021; Schiappa et al., 2018). In this context, lucid dreaming therapy represents a cognitive approach in which patients are trained to recognise and alter their dream content, particularly during nightmares, through specific exercises practiced while awake (Spoormaker & van den Bout, 2006; Zadra & Pihl, 1997). The induction of lucid dreams specifically aims to allow individuals to alter distressing dream imagery while still in the dream state, effectively converting nightmares into less frightening experiences. This peculiar form of dreaming may, in fact, provide an opportunity to increase self‐control and emotional regulation. In this sense, this ability to recognise and exert control over oneiric activity may contribute not only to a reduction in nightmare frequency but also to improvements in nightmare disorder and related sleep disturbances (de Macêdo et al., 2019; Hess et al., 2017). Moreover, lucid dreaming has also been associated with a range of other potential benefits, including problem‐solving, wish fulfillment, facilitating spiritual experiences and meditation, and promoting physical and mental healing (Schädlich & Erlacher, 2012; Stumbrys & Erlacher, 2016). Also, the positive relationship between poorer sleep quality and lucid dream frequency is consistent with existing literature. Studies indicate that poorer sleep quality often results in more fragmented sleep, which may facilitate lucid dreaming. This association aligns with the activation hypothesis (Hobson & McCarley, 1977) and the arousal‐retrieval model (Koulack & Goodenough, 1976), which propose that lighter, fragmented sleep enhances dream awareness and recall, with frequent awakenings reinforcing dream lucidity (Baird et al., 2019; Scarpelli et al., 2021). Lucid dreaming, specifically, is linked to lighter sleep characterised by partial arousals and increased brain activation, reflected by desynchronised EEG patterns that indicate heightened mental processing, including self‐awareness. Thus, when sleep is shallower and more fragmented—as with poorer sleep quality—this heightened arousal may facilitate awareness within the dream experience. Additional support for this relationship comes from studies demonstrating that stimulating the lower gamma band during REM sleep increases self‐awareness within dreams, effectively inducing lucid dreaming (Voss et al., 2014). Likewise, narcoleptic patients, who experience frequent awakenings and rapid transitions between sleep stages, report frequent lucid dreams and increased daytime sleepiness (Scarpelli et al., 2021), further supporting the idea that fragmented sleep naturally promotes lucidity (Dodet et al., 2015; Rak et al., 2015).

Finally, our findings suggested that women who indicated having unplanned pregnancies reported higher scores in nightmare distress. This finding is consistent with the idea that daytime worries and emotions have continuity in sleep (Domhoff, 2017). In this sense, the increased insecurity and fears associated with an unexpected pregnancy could, especially in the first trimester, heighten distress during oneiric activity. Furthermore, as mentioned previously, nightmares may play a role in emotional preparation for what is to come. In this vein, if there has not been adequate formation of maternal representations during the day, these can impact the emotional aspects of nightmares and affect psychological well‐being (Ammaniti et al., 2013; Lara‐Carrasco et al., 2013).

Overall, these findings extend the growing body of evidence that underscores the regulatory importance of dreaming during the period of pregnancy.

### 4.2 Characteristics of dream activity in alexithymic women during pregnancy

Our second hypothesis was to investigate differences in dream characteristics between pregnant women who exhibit alexithymic traits and those who do not, as well as to examine whether alexithymic traits moderate the relationship between depression, sleep quality, and dream characteristics. The regulatory function of dreaming is further highlighted when comparing non‐alexithymic women to alexithymic women, whose capacity for emotional regulation is impaired. Our findings indicate a significant relationship between alexithymic traits and both nightmare frequency and nightmare distress, aligning with previous research in the general population linking alexithymia to a greater frequency of distressing nightmares (Lumley & Bazydlo, 2000; Nielsen et al., 2011).

Considering that nightmares, but not dream recall frequency, were associated with alexithymia (i.e. difficulties in identifying feelings), it suggests that nightmares, rather than dreams in general, reflect failures in adaptive emotional regulation while awake, in line with previous research on the general population (Levin & Nielsen, 2009). Difficulties in identifying and describing feelings can lead to impaired emotional processing and regulation during the day (Godin et al., 2013). This emotional dysregulation may extend into sleep, manifesting as an increased frequency of nightmares and greater distress and emotional arousal (Lyvers et al., 2023; Nielsen et al., 2011). Thus, individuals with alexithymia may experience more intense and dysregulated emotional states, expressed in the form of nightmares. While during wakefulness, deficits in emotional regulation and the inability to cope with distressing emotions through mental processes can manifest as unexpected emotional outbursts (Taylor et al., 1999), during sleep, these same deficits in emotional regulation can interfere with the adaptive processes of dreaming, preventing the formation of creative and complex narratives (Bauermann et al., 2008; Lumley & Bazydlo, 2000; Nielsen et al., 2011). These findings suggest the hypothesis that nightmares and alexithymia may share a common neurobiological pathology related to emotional regulation during both sleep and wakefulness (Godin et al., 2015; Nielsen et al., 2011), as emerged in previous studies on individuals reporting traumatic experiences (Lee et al., 2021).

The evidence that nightmares reflect failures in adaptive emotional regulation is additionally supported when considering the interaction between the presence of depression and alexithymia. In the moderation analysis, in fact, depression predicted the frequency of nightmares only in women with higher levels of alexithymia. These findings are in line with a recent study showing the moderating effects of difficulties in emotional regulation on the relationship between mood disorders and nightmares in an inpatient psychiatric sample (Blanchard et al., 2024). Nightmare frequency is significantly higher in alexithymic women who also have higher depression scores. These women likely need to regulate their emotions, and this failure in regulation is probably reflected in the increased frequency of nightmares. In contrast, non‐alexithymic women and alexithymic women who do not report depressive symptomatology above the threshold, where the need for emotional regulation is presumably less pronounced, consistently exhibit lower frequencies of nightmares.

## 5 CONCLUSIONS

In summary, nightmare frequency appears to be an indicator of reduced regulatory capacity, particularly in alexithymic women with depressive symptoms. Given the ease with which nightmare frequency can be assessed, focussing on this aspect of dreaming could be an effective method for the early identification of pregnant women with impaired emotional regulation and higher levels of depressive symptoms. This is especially relevant considering the negative effects that emotional dysregulation and prepartum psychological distress can have during the postpartum period, as extensively demonstrated in the literature. Future studies should further investigate the contents of nightmares in alexithymic and non‐alexithymic and depressed and non‐depressed pregnant women.

The findings highlight the significant role of emotional regulation in the frequency and distress of nightmares, particularly in pregnant women with alexithymia and depression. These results suggest the need for targeted interventions focussing on improving emotional regulation in this vulnerable group of pregnant women.

Finally, this study is not without limitations. First, data on women's dreaming characteristics were collected through a self‐reported questionnaire. More detailed information on sleep quality and other sleep measures (such as nocturnal awakening frequency) could have been included to provide a better understanding of the relationship between the variables. Our aim was to explore the relationship between pregnancy‐related variables and dream characteristics, considering alexithymic traits. Consequently, we focussed on a sample of pregnant women. Future studies should extend this investigation by analysing whether similar patterns are observed in postpartum and non‐pregnant women.

## AUTHOR CONTRIBUTIONS

**Marta Spinoni:** Writing – original draft. **Serena Scarpelli:** Writing – review and editing. **Ilaria Di Pasquale Benedetti:** Writing – original draft. **Carlotta Med:** Writing – original draft. **Paola Ciolli:** Validation. **Francesco Rech:** Visualization. **Luigi De Gennaro:** Supervision. **Caterina Grano:** Writing – review and editing.

## FUNDING INFORMATION

The authors have no research funding to declare.

## CONFLICT OF INTEREST STATEMENT

Authors have no conflicts of interest to declare.

## PATIENT CONSENT

All participants took part voluntarily and were not remunerated. After explaining the study and obtaining online informed consent, women were asked to complete an online questionnaire.

## Data Availability

Research data are not shared.
